# Light on the cell cycle of the non-photosynthetic bacterium *Ramlibacter tataouinensis*

**DOI:** 10.1038/s41598-019-52927-7

**Published:** 2019-11-11

**Authors:** Gilles De Luca, Sylvain Fochesato, Jérôme Lavergne, Katrina T. Forest, Mohamed Barakat, Philippe Ortet, Wafa Achouak, Thierry Heulin, André Verméglio

**Affiliations:** 1 Aix Marseille Univ, CEA, CNRS, BIAM, LEMiRE, Saint Paul-Lez-Durance, F-13108 France; 2 Department of Bacteriology, University of Wisconsin-Madison, Madison, Wisconsin USA

**Keywords:** Bacteriology, Biophysical chemistry

## Abstract

*Ramlibacter tataouinensis* TTB310, a non-photosynthetic betaproteobacterium isolated from a semi-arid region of southern Tunisia, forms both rods and cysts. Cysts are resistant to desiccation and divide when water and nutrients are available. Rods are motile and capable of dissemination. Due to the strong correlation between sunlight and desiccation, light is probably an important external signal for anticipating desiccating conditions. Six genes encoding potential light sensors were identified in strain TTB310. Two genes encode for bacteriophytochromes, while the four remaining genes encode for putative blue light receptors. We determined the spectral and photochemical properties of the two recombinant bacteriophytochromes *Rt*BphP1 and *Rt*BphP2. In both cases, they act as sensitive red light detectors. Cyst divisions and a complete cyst-rod-cyst cycle are the main processes in darkness, whereas rod divisions predominate in red or far-red light. Mutant phenotypes caused by the inactivation of genes encoding bacteriophytochromes or heme oxygenase clearly show that both bacteriophytochromes are involved in regulating the rod-rod division. This process could favor rapid rod divisions at sunrise, after dew formation but before the progressive onset of desiccation. Our study provides the first evidence of a light-based strategy evolved in a non-photosynthetic bacterium to exploit scarse water in a desert environment.

## Introduction

The betaproteobacterium *Ramlibacter tataouinensis* TTB310 was previously isolated from sand particles coating a meteorite fragment collected from a semi-arid region of southern Tunisia (Tataouine) and identified as a new genus and species^1,2^. One of the most unusual characteristics of *R. tataouinensis* is its occurrence as both rod-shaped and spherical cells. The spherical cells are cysts, and are embedded within thick extracellular polymeric substances (EPS) that provide long-term desiccation resistance^2–4^. The rod-shaped cells are highly motile, which allows the colonization of a new location where they revert into cysts (Supplementary Fig. S1). In contrast to the classical cell cycle with a cyst stage that must differentiate into rods before division (*e.g*. *Azotobacter* and *Azospirillum*, among others), *R. tataouinensis* cysts are able to divide and separate when water and nutrients are available (Supplementary Fig. S1). These spherical cells capable of dividing are considered cysts because of their tolerance to desiccation^5^. Their ability to divide is compatible with their cyst status as demonstrated for the “reproductive cysts” of some free-living ciliates^6,7^. Cyst division is also observed in *Rhodospirillum centenum*, an anoxygenic photosynthetic and thermotolerant bacterium, when depleted of nutrients^8^. The daughter cells no longer divide 36–48 h after cyst induction. However, “hypercyst” mutants, which can readily form cysts on nutrient-rich medium, still cluster after cyst division^9^.

The unique cell cycle of *R. tataouinensis*, which alternates between desiccation-tolerant cysts capable of division and desiccation-sensitive motile rods capable of dissemination and division, is well suited for life in the hot and dry conditions of a desert environment. Due to the strong correlation between light, heat and desiccation, light should be an important external cue allowing *R. tataouinensis* to anticipate desiccation events by induction of protective mechanisms. In this context, six genes encoding potential light sensors were previously identified in strain TTB310^5^, representing one of the highest proportions of light-sensing proteins exhibited by a chemotrophic non-phototrophic bacterium^10^. Two of these genes (*Rta_25470* and *Rta_28950*) contain all of the hallmarks of a bacteriophytochrome, whereas the other four genes encode blue light receptors. Specifically, *Rta_12790* encodes a blue light-sensing histidine kinase containing a LOV (Light Oxygen Voltage) domain (phototropin analogous), while *Rta_31060*, *Rta_20590* and *Rta_26080* encode putative “Blue Light Using FAD” (or BLUF) proteins, although it has been noted that a reactive tyrosine and many highly conserved BLUF residues are absent from *Rta_26080*^10^.

The N-terminal photosensory input of phototropins containing two flavin mononucleotides (FMN) is coupled to a C-terminal output region that comprises a classic serine/threonine kinase motif. The blue light photoactivation of one of the two FMN induces protein rearrangements leading to autophosphorylation of the C-terminal kinase domain. BLUF photoreceptors bind flavin adenine dinucleotide (FAD) as chromophore. The output domains are PAS domains, GGDEF or EAL domains. Bacteriophytochromes possess a N-terminal photosensory core domain (PCD) that autocatalytically attaches a linear tetrapyrrole, whose photo-isomerization links two photo-interconvertible states that absorb in the red (Pr form) or far-red (Pfr form). The C-terminal module often contains a histidine kinase motif, involved in dimerization and signal transduction via phosphotransfer to a response regulator^11,12^.

The role of these light receptors has already been reported in several instances. In *Pseudomonas syringae*, a phototropin interacts with the bacteriophytochrome BphP1 to regulate swarming motility in response to light^13^. In *Rhodobacter sphaeroides*, photosystem synthesis is controlled by oxygen tension and light quantity. Both the BLUF protein AppA and the cryptochrome-like protein CryB are involved in this regulation^14,15^. The *Bacillus subtilis* phototropin YtvA is one of the stress input proteins that, when activated, leads to phosphorylation of parts of the macromolecular complex known as the stressosome^16,17^. It was also shown that infection by pathogenic bacteria is controlled by light via LOV domain histidine kinases in *Brucella abortus*^18,19^. More generally, it has been proposed that blue light receptors could regulate the transition between single and multicellular lifestyles^20^. Despite their large number in bacteria, the physiological role of most of these bacteriophytochromes remains unknown.

Far-red and red lights regulate the growth rate of cyanobacteria via the bacteriophytochromes Cph1 and Cph2, respectively^21^. Cph2 also inhibits phototaxis towards blue light^22^. In *Rhodopseudomonas palustris* and *Bradyrhizobium*, bacteriophytochromes regulate the synthesis of the photosynthetic machinery^23,24^. Carotenoid synthesis is regulated by a bacteriophytochrome in *Deinococcus radiodurans*^25^. A knockout mutant of the *Pseudomonas aeruginosa* bacteriophytochrome did not present a clear phenotype, although transcriptomic and proteomic analyses suggest the possible connection of bacteriophytochrome to quorum-sensing^26^. One of the two bacteriophytochromes present in *Azospirillum brasilense* Sp7 is required for the survival of on minimal medium under red light^27^. Its deletion increased sensitivity to the photooxidative stress^27^. In *Agrobacterium fabrum*, the conjugation is under the control of bacteriophytochromes^28^. More recently, it has been shown that bacteriophytochromes of *Pseudomonas syringae* pv. *tomato* were responsible for the repression of coronatine biosynthesis genes upon exposure to red light. This leads to the decrease of the stomatal reopening and bacteria entry^29^. Considering the fundamental signaling capability of light across all domains of life, and how extensively light-regulated pathways have been studied in animals, plants, and photosynthetic bacteria, we have only scratched the surface of light regulation in non-photosynthetic microbes. This and future studies are important for understanding a basic information processing problem.

In the present work, we studied the effect of light on *R. tataouinensis* TTB310, with a focus on the biochemical and biophysical properties of the two bacteriophytochromes and their roles in the cell cycle of this intriguing bacterium. Our results demonstrate that these two bacteriophytochromes initiate the rod division under red light, favoring a rapid division at sunrise before desiccation.

## Results

### Darkness, light and temperature effects on the growth of *R. tataouinensis* TTB310

**Figure 1:**
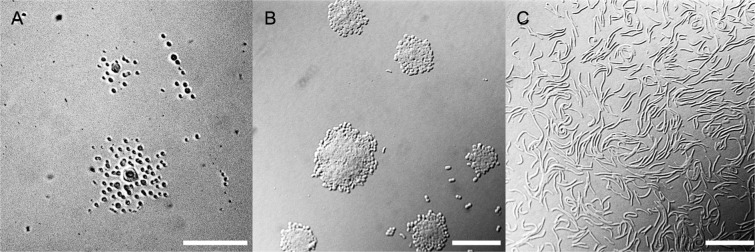
Morphology of *Ramlibacter tataouinensis* TTB310 grown in the dark or under red light illumination. (**A**) Image of *Ramlibacter tataouinensis* TTB310 grown in the dark on a Petri dish for 48 h at 25 °C (130x magnification). Scale bar: 200 µm. (**B**) Same as panel A but with 1000x magnification. Scale bar: 20 µm. (**C**) Cells subjected to continuous red light illumination for 48 h at 25 °C (18 µmol m^−2^ s^−1^; 1000x magnification). Scale bar: 20 µm.

Given the abundance of photoreceptors in strain TTB310, we sought to further characterize the role of light on growth in this bacterium. In a first set of experiments, we followed the development of strain TTB310 after 48–96 h of growth in darkness on agar plates at temperatures between 15 and 30 °C. Bacteria formed colonies composed essentially of cysts at all temperatures tested. However, at temperatures higher than 22 °C, the initial colony was surrounded by several equidistant colonies (Fig. 1A,B). This implies that a few mobile rod-shaped cells (emissaries) left the first colony to migrate and form new colonies, where a majority of cyst divisions occurs. Such rod-shaped cells have been observed at the periphery of colonies made of cysts^3^. Cell growth was monitored up to 7 days under continuous white light illumination provided by fluorescent tubes. Startlingly, we observed that relatively low-intensity (144 µmol m^−2^ s^−1^) white light illumination strongly inhibited the growth of strain TTB310 (Supplementary Table S1) in comparison to growth at the same temperature in the dark. This growth inhibition was partially reversible when cells were placed in the dark after exposure to the fluorescent light, as seen in previous experiments on day/night cycle exposure^5^. No effect was observed on the development of the *E. coli* control under the same illumination conditions. In contrast with our observation, we were expecting a high resistance to light in strain TTB310 that could be used to overcome the strong illumination prevailing in the desert.

To better understand the respective roles of the red/far-red and blue light receptors in this unexpected light inhibition, growth of strain TTB310 was monitored under red- or blue-filtered illumination. The light intensities were respectively 110 and 18 µmol m^−2^ s^−1^ for the resulting blue and red lights. Only the blue light induced light inhibition of growth (Supplementary Table S1), whereas the red light did not impair growth but did drastically increase rod division frequency (Fig. 1C). Similar results were obtained for illumination by red (660 nm) or far-red (780 nm) LEDs. In both cases, light induced the formation of a large number of motile rod-shaped cells that divided and disseminated on the agar surface, and only a few cysts were present after one week of observation. Varying the light intensity of the illumination at either 660 or 780 nm demonstrates that the induction of rod divisions occurs in both cases even at a very low intensity (0.5 µmole m^−2^ s^−1^).

These results suggest that blue light is responsible for growth inhibition of strain TTB310, whereas the induction of rod division could be mediated via light specifically absorbed by the bacteriophytochromes.

### Bacteriophytochrome characterization

**Figure 2:**
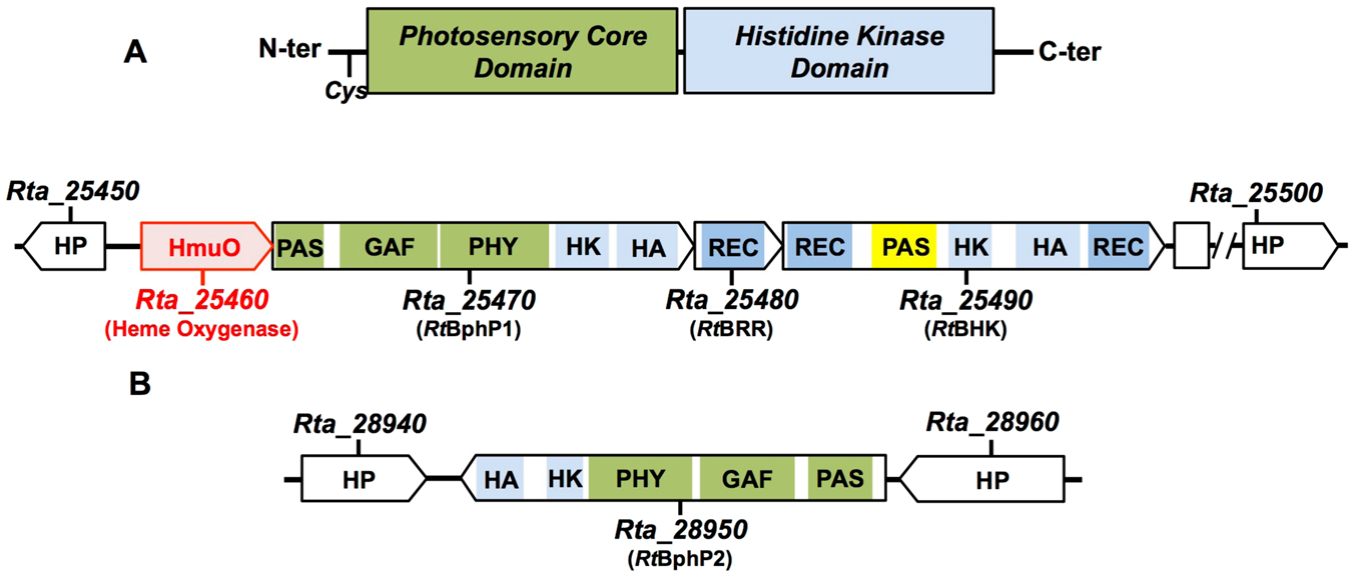
Bacteriophytochromes of *Ramlibacter tataouinensis* TTB310. (**A**) Schematic organization of bacteriophytochrome (BphP) proteins. The PCD contains PAS, GAF and PHY subdomains and a Cys residue located near the N-terminus that covalently binds the biliverdin chromophore. The Histidine Kinase signal transduction domain is located at the C-terminus of the protein. (**B**) Genetic context and structural organization of the *R. tataouinensis* bacteriophytochromes *Rt*BphP1 and *Rt*BphP2, and their cognate regulators (*Rt*BRR and *Rt*BHK). Abbreviations: HP: Hypothetical Protein; HmuO: Heme Oxygenase; PAS: Per-Arnt-Sim domain; GAF: cGMP-specific phosphodiesterases, Adenylyl cyclases and FhlA domain; PHY: Phytochrome domain; HK: HisKA (Histidine Kinase A phosphoacceptor) domain; HA: HATPase_c Histidine kinase-like ATPase, C-terminal domain; REC: Che Y-homologous regulator domain. *Rt*BRR: *R*. *tataouinensis* Bacteriophytochrome Response Regulator; *Rt*BHK: *R*. *tataouinensis* Bacteriophytochrome atypical hybrid Histidine Kinase.

Sequence analysis revealed that *Rt*BphP1 and *Rt*BphP2 display the classical bacteriophytochrome architecture with an N-terminal PCD and a C-terminal Histidine-Kinase Domain (Fig. 2A). They are phylogenetically distant with only 32% sequence identity and 36% similarity, respectively (Supplementary Fig. S2). The *Rta_25470* gene (*Rt*BphP1) belongs to a putative operon including a gene that encodes a heme oxygenase (*Rta_25460/hmuO*) and two potential cognate regulators (*Rta_25480/cheY* and *Rta_25490/atypical hybrid Histidine Kinase*) (Fig. 2B). The *Rta_28950* (*Rt*BphP2) gene is not associated with response regulator genes (Fig. 2B).

Due to the extreme growing conditions in the desert, the TTB310 bacteriophytochromes may present unusual photochemical properties, as already observed for several other bacteriophytochromes^24^. We therefore expressed and purified recombinant holo bacteriophytochromes of strain TTB310 to determine their spectral characteristics.

### Expression and purification of recombinant holobacteriophytochromes

The genes encoding *Rt*BphP1 and *Rt*BphP2 were co-expressed with a previously characterized heme oxygenase gene required for chromophore synthesis^30^ in *Escherichia* *coli* and subsequently purified by affinity chromatography. Gel electrophoresis and zinc fluorescence demonstrated that the biliverdin (BV) is covalently bound in both proteins, as previously reported for bacteriophytochromes of various bacterial species. Both bacteriophytochromes are dimeric, as indicated by cross-linking experiments (data not shown).

### Spectral properties

**Figure 3:**
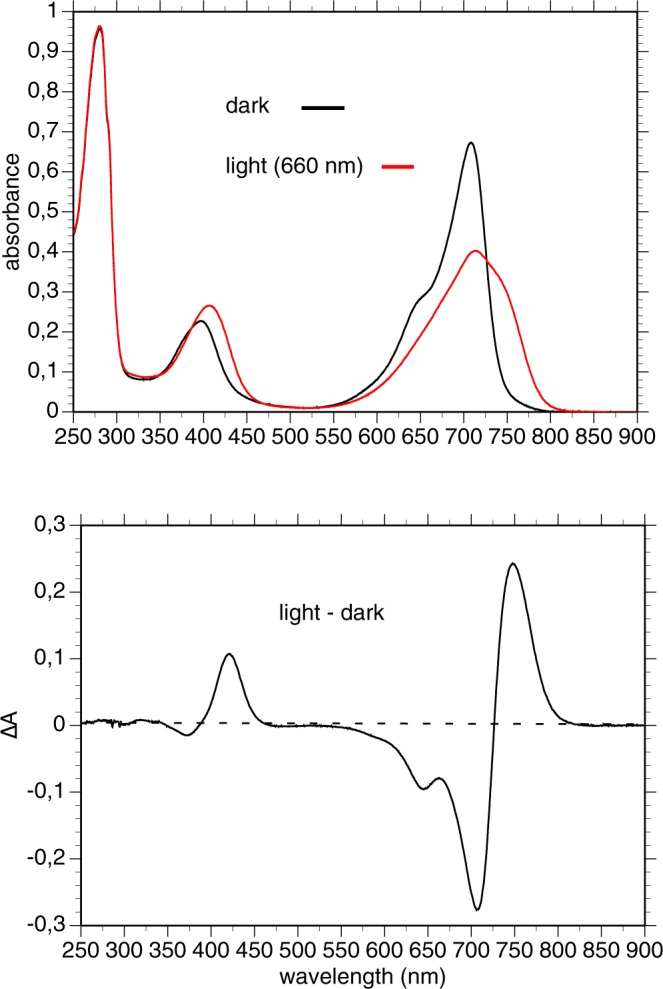
Spectral characterization of recombinant *Rt*BphP1. Top: absorption spectrum of a dark-adapted sample before (*black line*) and after (*red line*) photoconversion by 660-nm light. Bottom: light-dark difference spectrum.

**Figure 4:**
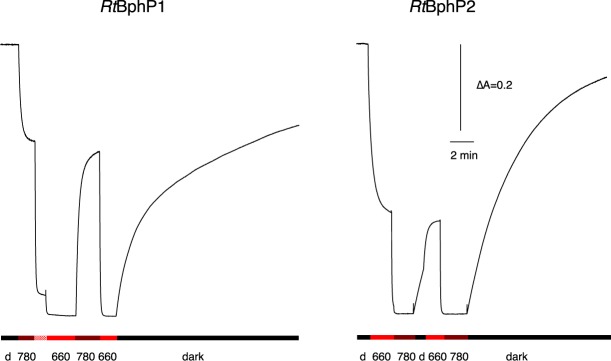
Light-induced kinetics for recombinant *Rt*BphP1 and *Rt*BphP2. Left: kinetics of light-induced absorbance changes measured at 705 nm for a dark-adapted suspension of *Rt*BphP1. The sample was successively subjected to 780-nm illumination (dark red), 780-nm + 660-nm illumination (checkered red light), 660-nm illumination (light red), 780-nm illumination, 660-nm illumination, and finally a long dark period. Right: kinetics of light-induced absorbance changes measured at 755 nm for a dark-adapted suspension of *Rt*BphP2. The sample was successively subjected to 660-nm illumination (light red), 780-nm illumination (dark red), a dark period (dark), 660-nm illumination (light red), 780-nm illumination (dark red), and finally a long dark period. The time and absorbance scales apply to both the left and right parts.

In agreement with the results of Baker *et al*.^31^ we observed, in a dark-adapted sample of purified recombinant *Rt*BphP1, an absorption spectrum characteristic of the Pr form of a *bona fide* BphP, with absorption maximum centered around 708 nm (Fig. 3, top, black line). Upon illumination with 660-nm light, a typical Pr/Pfr transition was observed with the large bleaching of the main absorption band and the appearance of a broad band centered at 740 nm (Fig. 3, top, red line). The light-minus-dark difference spectrum is typical for bacteriophytochromes (Fig. 3 bottom). Unexpectedly, illumination with a 780-nm light, a wavelength only very weakly absorbed by the Pr state of a dark-adapted sample of *Rt*BphP1 (Fig. 3, top, black line), induced the formation of approximately one-third of the full Pfr state. This is clearly shown in Fig. 4 (left) where we measured the light-induced absorbance changes at 705 nm following a 780-nm illumination of a dark-adapted sample of *Rt*BphP1. Illumination by both 780-nm and 660-nm lights induced approximately 90% of Pfr state of *Rt*BphP1. Complete transformation to the Pfr state was obtained by a 660-nm illumination (Fig. 4, left). Starting from this state, a 780-nm illumination rapidly restored the Pr state to level previously obtained after the first 780-nm illumination (Fig. 4, left). The dark reversion from the Pfr to the Pr state is a multiphasic process (Fig. 4, left), as previously reported by Baker *et al*.^31^. A complete reversion required more than 90 min. Since the extinction coefficient of the Pfr form at 780 nm is much larger than the Pr form, we concluded that the induction of the Pfr state by this illumination could only occur if the quantum yield for the Pr state is much higher than the Pfr state. After analyzing the effects of illumination by 640 or 780-nm light on the Pr state of *Rt*BphP1 (Fig. 4, left, Supplementary Fig. S3), we deduced, using the equations derived from Giraud *et al*.^32^, that the quantum yield of the transition from the Pr state to the Pfr state is 6–7 times greater than the light-induced reverse transition. Such a large difference in quantum efficiency between Pr and Pfr forms has already been observed for example for *At*BphP1 (or Agp1), a bacteriophytochrome present in *Agrobacterium tumefaciens*^33^. One consequence of this large difference in quantum yield between the two *Rt*BphP1 forms is that excitation by any wavelength between 550 and 800 nm induces a partial or complete formation of the Pfr state. Therefore, *Rt*BphP1 acts as a simple detector of a broad spectrum of red light.

**Figure 5:**
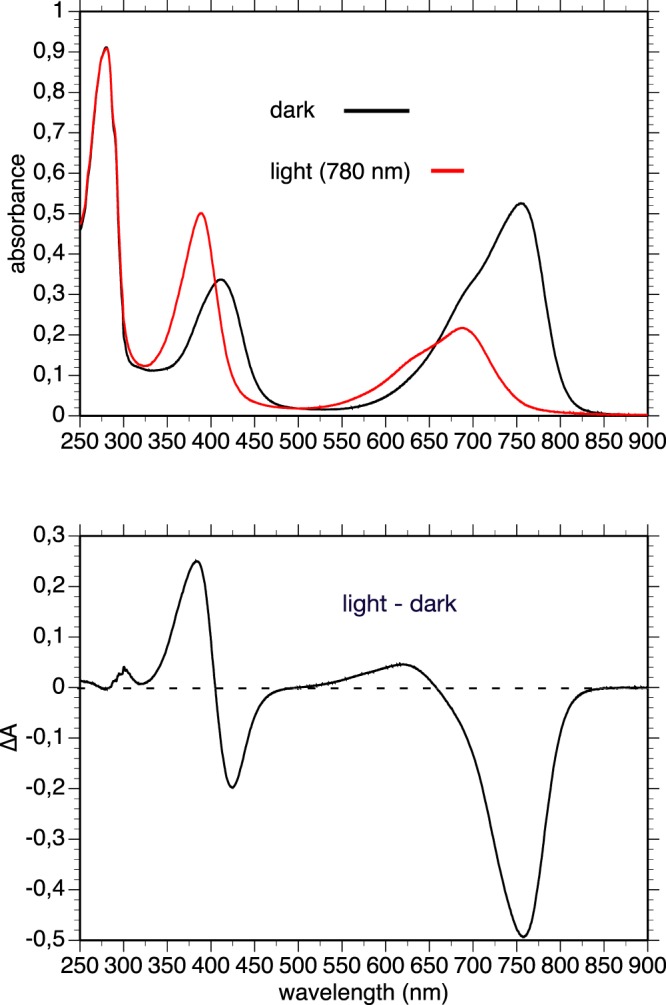
Spectral characterization of recombinant *Rt*BphP2. Top: absorption spectrum of a dark-adapted sample before (*black line*) and after (*red line*) photoconversion by 780-nm light. Bottom: light-dark difference spectrum.

The dark-adapted state of *Rt*BphP2 absorbs maximally at 755 nm, which is typical for a Pfr form (Fig. 5, top). Illumination of *Rt*BphP2 by infra-red light (780 nm) resulted in the rapid and complete formation of a Pr form with an absorption maximum centered at 690 nm (with an unusually low amplitude), whereas the amplitude of the Soret band was not affected (Fig. 5). Among the numerous phytochromes with a dark Pfr ground state, this peculiar property of the Pr form of *Rt*BphP2 was previously observed for the *A. tumefaciens* bacteriophytochrome *At*BphP2 (or Agp2)^34,35^ and for a truncated form of the *R. palustris* bacteriophytochrome *Rp*BphP1^36^. A 780-nm illumination completely bleached the 755-nm band, whereas a 660-nm illumination induced approximately two-thirds of the bleaching (Fig. 4, right). Since 660 nm is close to the isosbestic point of the light-dark difference spectrum of *Rt*BphP2 (Fig. 5, bottom), the formation of two-thirds of the Pr state by a 660-nm illumination implies that the quantum yield for the transition from Pfr to Pr for *Rt*BphP2 is about two times greater than the quantum yield of the reverse reaction. The reversion to the Pfr state occurs in the dark with a half-time of 3 min, and a 660-nm illumination slightly accelerates this reversion (Fig. 4, right). Due to the 2.5-fold larger extinction coefficient of the Pfr state as compared to the Pr state and the higher quantum yield of the Pfr state, any wavelength in the red and near infra-red induces a significant transition from the Pfr state to the Pr state. Like *Rt*BphP1, *Rt*BphP2 acts as a simple detector of a broad spectrum of red and far-red light.

### Phenotypes of mutants inactivated in heme oxygenase, bacteriophytochromes and cognate regulators

The unusual photochemical properties of *Rt*BphP1 and *Rt*BphP2 described above suggest that any wavelength of excitation in the red region, between 600 and 800 nm, will convert both *Rt*BphP1 and *Rt*BphP2 into a significant portion of their light-induced forms, respectively their Pfr and Pr forms. Therefore, we cannot predict whether only one bacteriophytochrome or both bacteriophytochromes are involved in the induction of rod divisions, based on the effect of the 660-nm or 780-nm illumination. To clarify their role in this differentiation process, we used plasmid insertion, the unique genetic tool that we have succeeded in developing in *R. tataouinensis*, without being able to complement the mutants without additional selective pressure (see Materials and Methods). Mutants inactivated for each of the bacteriophytochrome genes were constructed (∆*Rt*BphP1: *Rta*_25470 and ∆*Rt*BphP2: *Rta*_28950). Inactivation of the heme oxygenase gene (*∆Rt*HmuO: *Rta*_25460) and the atypical hybrid histidine kinase gene (∆*Rt*BHK: *Rta*_25490) was also achieved (Fig. 2B), whereas creation of a response regulator mutant (∆*Rt*BRR: *Rta*_25480) has failed despite multiple attempts.

**Figure 6:**
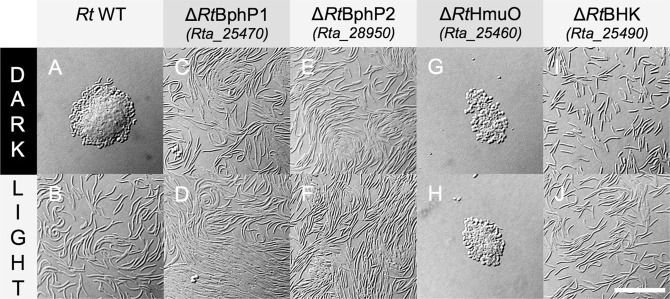
Morphology of *Ramlibacter tataouinensis* TTB310 wild type and mutants grown in the dark or under red light illumination. Images of *Ramlibacter tataouinensis* TTB310 grown for 48 h on Petri dishes. The WT and mutants were grown in the dark at 25 °C (**A**,**C**,**E**,**G**,**I**) or under continuous red illumination at 23 °C (**B**,**D**,**F**,**H**,**J**). Scale bar: 20 µm.

The heme oxygenase gene mutant (*∆Rt*HmuO) cannot synthesize the BV and therefore the two bacteriophytochromes cannot respond to light excitation. As expected, illumination of this mutant *∆Rt*HmuO with red light (660 or 780 nm) did not induce rod divisions (Fig. 6H), contrary to what was observed in the WT (Fig. 6B), and only cyst divisions were observed in both the dark and the light during the first 48 h of growth (Fig. 6G,H).

Inactivating the gene encoding *Rt*BphP1 or *Rt*BphP2 resulted in a surprising phenotype. Specifically, both mutants (∆*Rt*BphP1 and ∆*Rt*BphP2) displayed no cyst division in the dark as observed for WT but only motile rod divisions (Fig. 6C,E *vs*. A). Rod divisions were also observed under 660 or 780-nm illumination, as in the case of the WT (Fig. 6D,F *vs*. B). This phenotype could not result to plasmid excision due to the presence of kanamycin to maintain the selection pressure. We also exclude a potential polar effect on downstream gene expression since the genes of the heme oxygenase and of *Rt*BphP1 belong to the same operon, and nevertheless their mutant presented different phenotypes. Moreover, the phenotype linked to the inactivation of *Rt*BphP1 or *Rt*BphP2, which are genetically isolated, is similar. We are therefore confident in the fact that the phenotype observed for the mutants *Rt*BphP1 and *Rt*BphP2 are due to their inactivation.

The apparent contradiction between the evidence that light specifically absorbed by bacteriophytochromes induced rod divisions and the observation that their corresponding inactive mutant induced the same type of division, even in the dark, is considered in the Discussion section. We will also discuss the puzzling observation that mutation of one or the other of the two bacteriophytochromes induced the rod-rod division phenotype.

The phenotype of the atypical hybrid histidine kinase gene mutant (∆*Rt*BHK; *Rta*_25490) is similar to that of the two mutants ∆*Rt*BphP1 and ∆*Rt*BphP2, in that rod divisions occurred in the dark (Fig. 6I) and the light (Fig. 6J).

## Discussion

*R. tataouinensis* TTB310 presents a distinctive cell cycle and a complex network development on dilute nutrient agar. In the dark, the main process observed  is a cyst-cyst division (Supplementary Fig. S1 “cyst-cyst division”, Fig. 1A,B). Few motile rods, which derive  from peripheral cysts of the original colony, form distantly new colonies of cysts (Supplementary Fig. S1, “cyst-rod-cyst differentiation”)^5^. Illumination with white light of low intensity strongly inhibits the growth of *R. tataouinensis* TTB310 cells. This growth inhibition is specifically induced by blue light whereas red light leads to rod divisions (Supplementary Fig. S1 “rod-rod” division, Fig. 1C).

**Figure 7:**
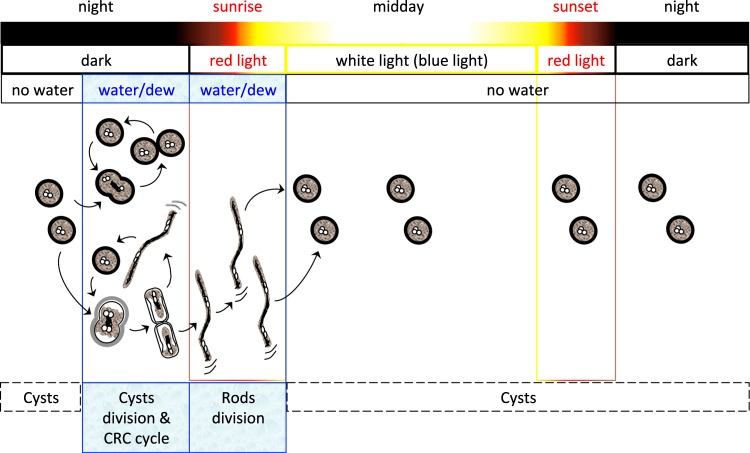
Hypothetical schema of light effect on the *Ramlibacter tataouinensis* cell cycle. It includes the cyst-rod-cyst differentiation cycle (CRC cycle), the cyst-cyst and rod-rod divisions, and the repression of the rod-rod divisions requiring the phosphorylation of bacteriophytochromes BphP1 and BphP2 in the dark.

Although the growth conditions we used in the present work did not replicate the desert environment, these particular effects of blue and red lights could provide some clues into the lifestyle of *R. tataouinensis* TTB310. In the desert, water is available essentially in the form of dew. This occurs when the temperature in the desert drops to the dew point of the ambient air, *i.e*. several hours before the end of the night. These conditions are met in the desert only a few days a year. In the dark and in the presence of water, cyst divisions and (less frequently) a cyst-rod-cyst cycle occurred (Figs 1 and 7). At sunrise (*i.e*. red light conditions), about one hour after dew formation, rod divisions are induced (Figs 1 and 7), allowing rapid cell division before the progressive increase of sunlight coupled to the onset of desiccation. Under these conditions, blue light from the sun should inhibit cell division and allow desiccation tolerance for those cells localized in the first millimeters below the sand surface (Fig. 7). This blue light inhibition probably involves one or several of the blue light receptors detected in the strain TTB310 genome. The precise mechanism of this regulation will be investigated in future studies. At sunset, the temperature of the sand is higher than the dew point of the ambient air. Therefore, despite the presence of red light, rod divisions should not occur due to the absence of water (Fig. 7).

The observation that rod division is stimulated by red light specifically absorbed by bacteriophytochromes demonstrates that these chromoproteins are involved in this regulation. The phenotype of the mutant inactivated for heme oxygenase, which does not exhibit the light-induced rod division, is in agreement with this conclusion.

The regulation of cyst formation by light is not restricted to *R. tataouinensis* TTB310. Deletion of Ppr, an unusual bacteriophytochrome present in *Rhodospirillum centenum*, inhibits the light regulation of a polyketide synthase gene (PKS)^37–39^ whose regulation is strongly correlated with stress-induced cyst formation^8^. Ppr, which acts as a simple detector of light^38,39^ as we describe here for *Rt*BPhP1 and *Rt*BPhP2, is therefore probably involved in the light regulation of cyst formation in *R. centenum*. Regulation of bacterial cyst formation by light, via bacteriophytochromes, could therefore be a general mechanism present in bacteria.

Analysis of the phenotype of the mutants *∆Rt*BphP1 and *∆Rt*BphP2 provides some insights to the mechanism of the regulation of cell cycle regulation by red light in *R. tataouinensis* TTB310. One intriguing result is the similar phenotype of these two mutants, for which rod division occurs in both the light and the dark. Since both *Rt*BphP1 and *Rt*BphP2 are both autophosphorylated in the dark^31^ (and data not shown), and this activity is partially suppressed by 700-nm light in the case of *Rt*BphP1^31^, we propose that the two autophosphorylated bacteriophytochromes repress rod division in the dark by transferring their phosphate to their response regulators (Fig. 7). This repression of rod divisions results in cyst division and the cyst-rod-cyst cycle (including the presence of rods generated from cyst differentiation) (Fig. 6A). In the light, the bacteriophytochromes are dephosphorylated. The response regulator cannot be phosphorylated and the repression of rod division can no longer occur (Figs 6B and 7), so rod divisions become possible. In the absence of either bacteriophytochrome (*∆Rt*BphP1 or *∆Rt*BphP2) in the dark as well as in the light, phosphorylation of the response regulator cannot occur, resulting in rod divisions (Figs 6C–F and 7). In the absence of the BV (by inactivation of the heme oxygenase gene, *∆Rt*HmuO mutant), the two bacteriophytochromes are phosphorylated both in the dark and in the light, and rod division is repressed under both conditions (Figs 6G,H and 7).

Although the mechanism described above explains the phenotype of *∆Rt*BphP1 or *∆Rt*BphP2 mutants, explaining the rod-rod division phenotype when only one of the two bacteriophytochromes is inactivated requires their direct or indirect cooperation. This raises two possibilities. In the first situation (direct cooperation), the active *in vivo* bacteriophytochrome is a heterodimer of *Rt*BphP1 and *Rt*BphP2, phosphorylated in the dark. In the absence of *Rt*BphP1 or *Rt*BphP2 (*i.e*. in *∆RtBphP1* or *∆RtBphP2* mutants), the active heterodimer cannot be formed, *Rt*BRR cannot be phosphorylated in the dark, and rods are the dominant phenotype of these mutants (Figs 6I and 7). Interestingly, the presence of phytochrome heterodimers has been clearly demonstrated in higher plants^40,41^. For example, phytochromes PhyC and PhyB form heterodimers in rice^41^ and *Arabidopsis thaliana*^40^. In rice, when PhyB is absent, PhyC exists as a monomer that is spectrophotometrically active but biologically inactive^41^. To test the possibility of heterodimerization between *Rt*BphP1 and *Rt*BphP2, we coexpressed their two genes in tandem in *E. coli* together with the heme oxygenase gene, but detected no bacteriophytochrome heterodimers formed in these conditions when our standard metal affinity chromatography protocol was carried out (data not shown). Although this experiment does not completely exclude the possibility of heterodimer formation *in vivo*, we do not favor this first hypothesis.

The second hypothesis is to consider an indirect cooperation between the two bacteriophytochromes via the cognate response regulator *Rt*BRR (Supplementary Fig. S4). In a first step, *Rt*BphP1 phosphorylates *Rt*BRR in the dark^31^. Due to the “arm-in-arm” arrangement of the *Rt*BRR dimer^31^, this phosphorylation could induce a conformational change in the second monomer of the dimer. After this conformational change, the second monomer can only be phosphorylated by *Rt*BphP2. This hypothesis takes into account the unusual “arm-in-arm” ultrastructure of *Rt*BBR and the absence of RR gene in the vicinity of the *Rta*_28950 gene (*Rt*BphP2) (Fig. 2B). In a more complex situation, each bacteriophytochrome reacts with its own response regulator. In this case, the gene regulation requires the cooperation of these two regulators possibly via the atypical hybrid histidine kinase *Rt*BHK, whose inactivation leads to the same phenotype as bacteriophytochrome inactivation (Fig. 6I,J).

In conclusion, our results establish that the two bacteriophytochromes of *R. tataouinensis* TTB310 are involved in the rapid rod division phase of the cell cycle. The cooperation of their signaling pathway and their particular optical properties constitutes a strategy that can be exploited to significantly broaden their efficiency from 550 to 800 nm. This mechanism, which occurs at sunrise while dew remains on sand particules, may favor a rapid dissemination of rods in order to avoid the noxious effects of strong light and dehydration. This is the first demonstration that (red) light controls the cell cycle of a non-photosynthetic bacterium, a phenotype likely coupled to this microbe’s tolerance to water scarcity. This is likely a complementary system to the internal circadian rhythm (the *kaiC* gene was detected in the genome of strain TTB310^5^) using the light as external cue.

Recently, a report has highlighted the roles of photoreceptors of *Pseudomonas syringae* pv. *tomato* in optimizing plant leaf invasion as a function of lighting conditions^29^. Involvement of photoreceptors in optimizing the development, dissemination and growth of bacteria as a function of the presence and quality of light may be a more general trait than previously anticipated.

## Materials and Methods

### Bacterial strains and growth conditions

*Ramlibacter tataouinensis* TTB310^T^^2^ was cultured at 30 °C in the dark in tenfold diluted tryptic soy broth (TSB/10, Difco Laboratories) or tryptic soy agar (TSA/10). *Escherichia coli* strains were cultured at 37 °C in LB medium or 30 °C on TSA/10 (conjugation experiments) with the appropriate antibiotic [Kanamycin (Km) 50 µg mL^−1^, Chloramphenicol (Cm) 50 µg mL^−1^, Ampicillin (Amp) 50 µg mL^−1^, or Streptomycin (Sm) 100 µg mL^−1^] and diaminopimelic acid (DAP) when necessary.

### Growth under various illumination systems

Strain TTB310 was cultured in TSB/10. After incubation at 30 °C for 72 h with shaking in the dark, bacteria were spread on TSB/10 agar plates (1.5 g L^−1^) and cultured at 23–30 °C in the dark, or in continuous light conditions in an incubator equipped with fluorescent lamps (Infors Multitron 2). Continuous light was provided by either cool white fluorescent lamps (SYLVANIA GRO-LUX® GRO/WS) filtered with blue or red/infra-red filters, or LEDs (660, 700, or 780 nm). For the light effect on growth of TTB310 (Supplementary Table S1), bacterial growth was estimated five times from serial dilution of a preculture grown in the dark with shaking (TSB/10, 30 °C) and from the number of colonies developing from each 5 µL drop (4 replicate drops for each dilution) deposited and cultivated at 22 °C on TSA/10 under different fluorescent lighting conditions (No Filter, Blue Filter, or Red filter) compared to the 100% control in dark conditions (using two layers of aluminum foil): the 100% control is equal to 2.10^7^ bacteria.mL^−1^.

### Morphological characterization of bacteria

Wild type (WT) and various mutants were observed with an inverted microscope (Willowert Wetzlar 21-D6330, X4 objective), and photographed (X10 objective) with a BX50 Olympus microscope equipped with a differential interference contrast (DIC) device and a digital camera (Olympus F-view II). When necessary, a small piece of agar supporting several colonies was covered with a glass cover slip, cut and placed on a microscope slide to observe the presence of cysts or rods with the 100 X oil immersion objective (UPlanApo, Olympus)^4^.

### Expression and protein procedures

The *Rta*_25470 and *Rta*_28950 genes, designated respectively as *Rt*BphP1 and *Rt*BphP2, were PCR-amplified from genomic DNA with the Herculase II Fusion DNA polymerase (Agilent Technologies) using primers designed to add appropriate restriction sites (Supplementary Table S2) for expression as His_6_-tagged versions in the pBAD/HisB expression vector (Invitrogen). An upstream alternative start codon that elongates the protein N-terminal sequence containing the presumed cysteine ligand of the biliverdin chromophore, which was absent from the previous annotation, was used to begin amplification of the *Rta*_25470 gene. These fragments were cloned into the pTOPO-XL cloning vector and sequence-confirmed, before digestion with the restriction endonucleases *Bgl*II/*Eco*RI for *Rt*BphP1 or *Bam*HI/*Eco*RI for *Rt*BphP2 (which already contains a *Bgl*II restriction site). Fragments were subsequently cloned into the pBAD/HisB-RpBphP2-HmuO plasmid [containing the *hmuO* gene that encodes the *Bradyrhizobium sp. ORS278* heme oxygenase^30^], predigested with the *Bgl*II/*Eco*RI endonucleases, and inserted in place of the *Rp*BphP2 gene. Overnight cultures of *E*. *coli* TOP10 containing pBAD/HisB-*Rt*BphP1-HmuO or pBAD/HisB-*Rt*BphP2-HmuO were grown with shaking (200 rpm) at 37 °C in LB medium supplemented with ampicillin (50 µg mL^−1^). The culture was diluted 1:100 into fresh LB medium with ampicillin 50 µg mL^−1^ (1 L in a 2-L flask) and grown at 37 °C with shaking (200 rpm) until an OD_600_ of 0.6 was obtained, at which point L-arabinose was added to a final concentration of 0.66 mM (0.01%). Protein expression was allowed to proceed overnight (16–18 h), with shaking at 30 °C before harvesting the cells by centrifugation (6,000 g for 5 min at 4 °C). The cell pellet, generally green in color, was used immediately or stored at −20 °C until initiation of protein purification. Cells were resuspended in buffer A (Hepes 10 mM pH 7.0 or Tris-HCl 50 mM pH 8.0, 0.5 M NaCl, 20 mM imidazole) with the addition of DNase I (10 mg mL^−1^), and broken by French Press lysis (2 kbar or 200 MPa) or by using a One Shot cell disruptor (CellD). The cell lysate containing the soluble holo-BphP was then clarified by centrifugation (6,600 g for 30 min at 4 °C) to remove insoluble material and cell debris, followed by a 0.22-µm filtration. The soluble fraction was applied onto a 1-mL HisTrap HP column (GE Healthcare) pre-equilibrated against buffer A and washed with 10 mL buffer A. Green-colored proteins were eluted with 2.5 mL buffer B (Hepes 10 mM pH 7.0 or Tris-HCl 50 mM pH 8.0, 0.5 M NaCl, 0.5 M imidazole) and immediately eluted with Tris-HCl 50 mM pH 8.0, 0.25 M NaCl using a PD-10 Desalting Column (GE Healthcare).

### Construction of mutants

*R. tataouinensis* is a new bacterial model system for which elaborate genetic tools were not yet available. The ability to construct the single gene knockout mutants presented in this work is a major step forward and these are the first mutants to be reported for *R. tataouinensis*. As part of developing genetic tools specific to *R*. *tataouinensis* TTB310, resistance to several antibiotics was examined. Only the broad host range pBBR1MCS-2-Km^r^ plasmid^42^ was successfully introduced in electrocompetent TTB310 cells and detected by the acquisition of kanamycin resistance. None of the other tested pBBR1MCS derivatives (Cm^r^, Sm^r^/Sp^r^, Amp^r^, Tet^r^, Gm^r^) conferred any antibiotic resistance (data not shown). Furthermore, preliminary attempts to inactivate a targeted gene in strain TTB310 by marker exchange mutagenesis failed, but did result in a plasmid integration that could be detected by the acquisition of kanamycin resistance encoded on the plasmid (data not shown). Therefore, we constructed a plasmid for all mutants that was unable to replicate in strain TTB310. This plasmid, pSW23T-Km^r^ (derived from pSW23T^43^, modified in this study with the insertion of a PCR-amplified kanamycin resistance encoding gene from pBBR1MCS-2, cloned at the *Bam*HI site), contains a fragment internal to the target gene whose integration into the chromosomal copy should disrupt the gene, resulting in a non-functional protein. Fragments of the genes *Rt*HmuO (*Rta*_25460; 0.5 kb), *Rt*BphP2 (*Rta*_28950; 1.1 kb), *Rt*BphP1 (*Rta*_25470; 0.3 kb), *Rt*BRR (*Rta*_25480 RR; 0.26 kb), and *Rt*BHK (*Rta*_25490 HK; 0.32 kb) were PCR-amplified from genomic DNA using the Herculase II Fusion DNA polymerase (Agilent Technologies) and primers listed in Supplementary Table S2. These fragments were then cloned into the pTOPO-XL cloning vector (Thermo Fisher Scientific). After sequence confirmation, each fragment was digested and cloned at the *Xba*I site of pSW23TKm^r^, except for *Rt*BHK fragment, which was cut from the two *Eco*RI sites flanking the pTOPO-XL multiple cloning site, and cloned at the *Eco*RI site of pSW23TKm^r^. The resulting ligated plasmids were transferred to the *E*. *coli* strain S17–1 λpir (Sm^r^). They were then purified and transferred by transformation to *E*. *coli* strain β3914, which is auxotrophic for DAP (Km^r^, Em^r^, Tc^r^)^44^, and transferred to strain TTB310 by overnight conjugation at 30 °C on TSA/10 complemented with DAP. The conjugation drop was made by resuspending a 10-mL pellet from a 24–72 h TTB310 culture in 200 µL TSB/10, and then using 75 µL of this solution to resuspend the pellet of 1-mL *E*. *coli* overnight culture. Transconjugants were subcultured on TSA/10 without DAP and selected with Km (200 µg mL^−1^). Km-resistant *R. tataouinensis* exconjugants were confirmed by PCR-amplification between plasmidic Km^r^ and the surrounding chromosomal region neighboring the PCR-amplified fragment, and the insertion site was sequence-confirmed for each mutant.

### Absorbance measurements

Purified bacteriophytochrome spectra were recorded with a Cary 50 spectrophotometer either in the dark or under continuous illumination (at 640, 660, 700 or 780 nm) provided by LEDs with irradiance between 60–70 µmol m^−2^ s^−1^. Light-induced absorbance changes were performed with a laboratory-built spectrophotometer^45^ similar to the one developed by Joliot *et al*.^46^. The absorption level was sampled using monochromatic flashes emitted 1 ms to several seconds after actinic excitation, provided by a 2-µs Xenon flash.

### Construction of the phylogenetic tree

As the two *Rt*BphPs and their 100-most homologous sequences are histidine kinases (sensor part of two-component system), phylogenetic analysis was based on the alignment of the Photosensory Core Domain (PCD) extracted from the P2CS database^47^. When homologous intra-species sequences were detected, only one was kept to make a simplified tree. This tree was generated by using the “*one click mode”* on phylogeny.fr website^48^.

## Supplementary information

Supplementary Information

## Data Availability

Almost all data generated or analysed during this study are included in this published article (and its Supplementary Information files). Raw data are available from authors upon request.

## References

[1] Gillet P (2000). Terrestrial biological activity in the Tatahouine meteorite. Clues for the search of nanometric life traces in rocks. Earth Plan. Sci. Lett..

[2] Heulin T (2003). *Ramlibacter tataouinensis* gen. nov., sp. nov., and *Ramlibacter henchirensis* sp. nov., cyst-producing bacteria isolated from sub-desert soil in Tunisia. Int. J. Syst. Evol. Microbiol.

[3] Benzerara K (2004). Experimental colonization and alteration of orthopyroxene by the pleiomorphic bacteria *Ramlibacter tataouinensis*. Geomicrobiol. J..

[4] Gommeaux M, Barakat M, Lesourd M, Thiery J, Heulin T (2005). A morphological transition in the pleiomorphic bacterium *Ramlibacter tataouinensis* TTB310. Res. Microbiol..

[5] De Luca G (2011). The cyst-dividing bacterium *Ramlibacter tataouinensis* TTB310 genome reveals a well-stocked toolbox for adaptation to a desert environment. PLoS ONE.

[6] Gabe PR, De Bault LE (1973). Macromolecular syntheses related to the reproductive cyst of *Tetrahymena patula*. J. Cell Biol..

[7] Long H, Zufall RA (2010). Diverse modes of reproduction in the marine free-living ciliate *Glauconema trihymene*. BMC Microbiol..

[8] Berleman JE, Bauer CE (2004). Characterization of cyst formation in the purple photosynthetic bacterium *Rhodospirillum centenum*. Microbiology.

[9] Berleman JE, Hasselbring BM, Bauer CE (2004). Hypercyst mutants in *Rhodospirillum centenum* identify regulatory loci involved in cyst cell differentiation. J. Bacteriol..

[10] Mandalari C, Losi A, Gärtner W (2013). Distance-tree analysis, distribution and copresence of bilin- and flavin-binding prokaryotic photoreceptors for visible light. Photochem. Photobiol. Sci..

[11] Rockwell NC, Su YS, Lagarias JC (2006). Phytochrome structure and signaling mechanisms. Annu. Rev. Plant Biol..

[12] Karniol B, Wagner JR, Walker JM, Vierstra RD (2005). Phylogenetic analysis of the phytochrome superfamily reveals distinct microbial subfamilies of photoreceptors. Biochem. J..

[13] Wu L, McGrane RS, Beattie GA (2013). Light regulation of swarming motility in *Pseudomonas syringae* integrates signaling pathways mediated by a bacteriophytochrome and a LOV protein. mBio.

[14] Masuda S, Bauer CE (2002). AppA is a blue light photoreceptor that antirepresses photosynthesis gene expression in *Rhodobacter sphaeroides*. Cell.

[15] Metz S (2012). Interaction of two photoreceptors in the regulation of bacterial photosynthesis genes. Nucleic Acids Res..

[16] Avila-Perez M, Hellingwerf KJ, Kort R (2006). Blue light activates the sigmaB-dependent stress response of *Bacillus subtilis* via YtvA. J. Bacteriol..

[17] Gaidenko TA, Kim TJ, Weigel AL, Brody MS, Price CW (2006). The blue-light receptor YtvA acts in the environmental stress signaling pathway of *Bacillus subtilis*. J. Bacteriol..

[18] Swartz TE (2007). Blue-light-activated histidine kinases: two-component sensors in bacteria. Science.

[19] Tseng TS, Frederickson MA, Briggs WR, Bogomolni RA (2010). Light-activated bacterial LOV-domain histidine kinases. Methods Enzymol..

[20] Gomelsky M, Hoff WD (2011). Light helps bacteria make important lifestyle decisions. Trends Microbiol..

[21] Fiedler B (2004). Involvement of cyanobacterial phytochromes in growth under different light qualities and quantities. Photochem. Photobiol..

[22] Wilde A, Fiedler B, Börner T (2002). The cyanobacterial phytochrome Cph2 inhibits phototaxis towards blue light. Mol. Microbiol..

[23] Giraud E (2002). Bacteriophytochrome controls photosystem synthesis in anoxygenic bacteria. Nature.

[24] Giraud E, Verméglio A (2008). Bacteriophytochromes in anoxygenic photosynthetic bacteria. Photosynth. Res..

[25] Davis SJ, Vener AV, Vierstra RD (1999). Bacteriophytochromes: phytochrome like photoreceptors from non-photosynthetic eubacteria. Science.

[26] Barkovits K, Schubert B, Heine S, Scheer M, Frankenberg-Dinkel N (2011). Function of the bacteriophytochrome BphP in the RpoS/Las quorum-sensing network of *Pseudomonas aeruginosa*. Microbiology.

[27] Kumar S (2012). Bacteriophytochrome controls carotenoid-independent response to photodynamic stress in a non-photosynthetic rhizobacterium, Azospirillum brasilense Sp7. Sci. Rep..

[28] Bai Y, Rottwinkel G, Feng J, Liu Y, Lamparter T (2016). Bacteriophytochromes control conjugation in *Agrobacterium fabrum*. J. Photochem. Photobiol. B.

[29] Santamaria-Hernando S (2018). *Pseudomonas syringae* pv. tomato exploits light signals to optimize virulence and colonization of leaves. Environ. Microbiol..

[30] Giraud E (2005). A new type of bacteriophytochrome acts in tandem with a classical bacteriophytochrome to control the antennae synthesis in *Rhodopseudomonas palustris*. J. Biol. Chem..

[31] Baker AW, Satyshur KA, Moreno Morales N, Forest KT (2016). Arm-in-arm response regulator dimers promote intermolecular signal transduction. J. Bacteriol..

[32] Giraud E, Lavergne J, Verméglio A (2010). Characterization of bacteriophytochrome from photosynthetic bacteria: histidine kinase signaling triggered by light and redox sensing. Methods Enzymol..

[33] Lamparter T, Michael N, Mittmann F, Esteban B (2002). Phytochrome from *Agrobacterium tumefaciens* has unusual spectral properties and reveals an N-terminal chromophore attachment site. Proc. Natl. Acad. Sci. USA.

[34] Krieger A, Molina I, Oberpichler I, Michael N, Lamparter T (2008). Spectral properties of phytochrome Agp2 from *Agrobacterium tumefaciens* are specifically modified by a compound of the cell extract. J. Photochem. Photobiol. B.

[35] Oberpichler I, Molina I, Neubauer O, Lamparter T (2006). Phytochromes from *Agrobacterium tumefaciens*: Difference spectroscopy with extracts of wild type and knockout mutants. FEBS Lett..

[36] Bellini D, Papiz MZ (2012). Structure of a bacteriophytochrome and light-stimulated protomer swapping with a gene repressor. Structure.

[37] Kyndt JA (2010). Regulation of the Ppr histidine kinase by light-induced interactions between its photoactive yellow protein and bacteriophytochrome domains. Biochemistry.

[38] Kyndt JA, Meyer TE, Cusanovich MA (2004). Photoactive yellow protein, bacteriophytochrome, and sensory rhodopsin in purple phototrophic bacteria. Photochem. Photobiol. Sci..

[39] Jiang ZY (1999). Bacterial photoreceptor with similarity to photoactive yellow protein and plant phytochromes. Science.

[40] Clack T (2009). Obligate heterodimerization of *Arabidopsis* phytochromes C and E and interaction with the PIF3 basic Helix-Loop-Helix transcription factor W. Plant Cell.

[41] Xie X, Kagawa T, Takano M (2014). The phytochrome B/phytochrome C heterodimer is necessary for phytochrome C-mediated responses in rice seedlings. PLoS ONE.

[42] Kovach ME (1995). Four new derivatives of the broad-host-range cloning vector pBBR1MCS, carrying different antibiotic-resistance cassettes. Gene.

[43] Demarre G (2005). A new family of mobilizable suicide plasmids based on broad host range R388 plasmid (IncW) and RP4 plasmid (IncPalpha) conjugative machineries and their cognate *Escherichia coli* host strains. Res. Microbiol..

[44] Le Roux F, Binesse J, Saulnier D, Mazel D (2007). Construction of a *Vibrio splendidus* mutant lacking the metalloprotease gene *vsm* by use of a novel counterselectable suicide vector. Appl. Environ. Microbiol..

[45] de Rivoyre M, Ginet N, Bouyer P, Lavergne J (2010). Excitation transfer connectivity in different purple bacteria: A theoretical and experimental study. Biochim. Biophys. Acta.

[46] Joliot P, Béal D, Frilley B (1980). Une nouvelle méthode spectrophotométrique destinée à l'étude des réactions photosynthétiques. J. Chim. Phys..

[47] Ortet P, Whitworth DE, Santaella C, Achouak W, Barakat M (2015). P2CS: updates of the prokaryotic two-component systems database. Nucleic Acids Res..

[48] Dereeper A (2008). Phylogeny.fr: robust phylogenetic analysis for the non-specialist. Nucleic Acids Res..

