# Supplementary material for: Light on the cell cycle of the non-photosynthetic bacterium Ramlibacter tataouinensis
Source: Sci Rep. 2019 Nov 11;9:16505. doi: 10.1038/s41598-019-52927-7 (PMC6848086; doi:10.1038/s41598-019-52927-7)
Supplement: Supplementary file 1 — Supplementary Information [file 41598_2019_52927_MOESM1_ESM.pdf]

Supplementary Information:

Light on the cell cycle of the non-photosynthetic bacterium *Ramlibacter tataouinensis*

Gilles De Luca[a], Sylvain Fochesato[a], Jérôme Lavergne[a], Katrina T. Forest[b], Mohamed Barakat[a], Philippe Ortet[a], Wafa Achouak[a], Thierry Heulin[a,1,#] & André Verméglio[a,1]

[a] *Aix Marseille Univ, CEA, CNRS, BIAM, LEMiRE, Saint Paul-Lez-Durance, France*

[b] *Department of Bacteriology, University of Wisconsin-Madison, Madison, Wisconsin, USA*

[1] T.H and A.V. contributed equally to this work.

E-mail address for correspondence: thierry.heulin@cea.fr

TABLE S1: **Light effect on growth of *Ramlibacter tataouinensis***

Bacteria growth was estimated from serial dilution of a preculture grown in the dark with shaking (TSB/10, 30 °C) and from the number of colonies developing from each 5 μL drops deposited and grown on TSA/10 under different lighting conditions at 22 °C. 100% = 2. 10^{7} bacteria.mL^{-1}.

**Culture Conditions**	**Growth Percentage**
Dark (control)	100%
Fluorescent Light (No Filter)	<0.01 to 0.1%
Blue Light (Filtered Fluorescent Light)	0.01 to 0.1%
Red Light (Filtered Fluorescent Light)	100%

TABLE S2: List of oligonucleotide primers used

Use	*Name*	*Primer sequence*
Bacteriophyto-chromes expression	*Rt*BphP1 F (*Bgl*II)	5'-AGATCTATGAACCTTCCGCCGCCTGA-3'
	*Rt*BphP1 R (*Eco*RI)	5'-GAATTCTTAAGCATGGTTCCTGTCCTCT-3'
	*Rt*BphP2 F (*Bam*HI)	5'-GGATCCATGTACGCCACTTTCACCAGC-3'
	*Rt*BphP2 R (*Eco*RI)	5'- GAATTCCTACCGCCGCGTCGGCCA-3'
Insertional mutagenesis	*Rt*HmuO F (*Xba*I)	5'- TCTAGAAGGTCGTTGGAACAACAGCCC-3'
	*Rt*HmuO R (*Xba*I)	5'-TCTAGACGTGGTCACGTTGCGTTCCA-3'
	*Rt*BphP2 F (*Xba*I)	5'-TCTAGAGCCGCCATGCTGATCGTCTG-3'
	*Rt*BphP2 R (*Xba*I)	5'-TCTAGAGGCTTCGACCGGGTCATGG-3'
	*Rt*BphP1 F (*Xba*I)	5'- TCTAGAACCTGGTGGTGGAGTTCGAG-3'
	*Rt*BphP1 R (*Xba*I)	5'-TCTAGAGTAGTTGGCATCCGGGATCAG-3'
	*Rt*BRR (*Rta*_25480) F (*Xba*I)	5'-TCTAGAAGCGCAGCAAGCTCTCCAAC-3'
	*Rt*BRR (*Rta*_25480) R (*Xba*I)	5'-TCTAGAGCCCAGCTCGTAACTCTTGAC-3'
	*Rt*BHK (*Rta*_25490) F (*Xba*I)	5'-TCTAGAAGCTGCGCACGCCGCTCAC-3'
	*Rt*BHK (*Rta*_25490) R (*Xba*I)	5'-TCTAGAGCTGGATGCGCGCCTCGTC-3'

FIGURE S1

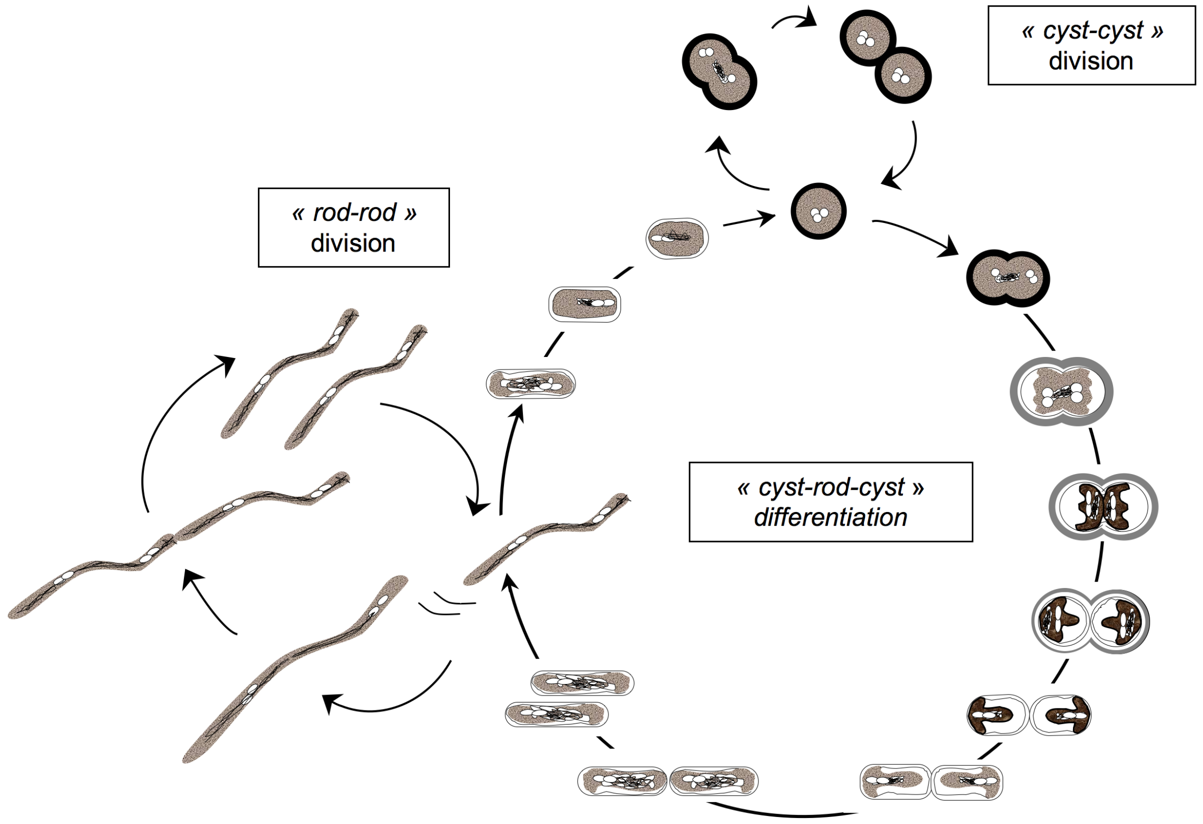

FIGURE S1. Cell cycle of *Ramlibacter tataouinensis* TTB310

The life cycle includes the cyst-to-cyst division step (*cyst-cyst* division) and the cyst-to-rod division step, plus the reversion from the rod-to-cyst component of the cycle (*cyst-rod-cyst* differentiation). The rod-to-rod division step is included as a step in the *cyst-rod-cyst* differentiation.

FIGURE S2

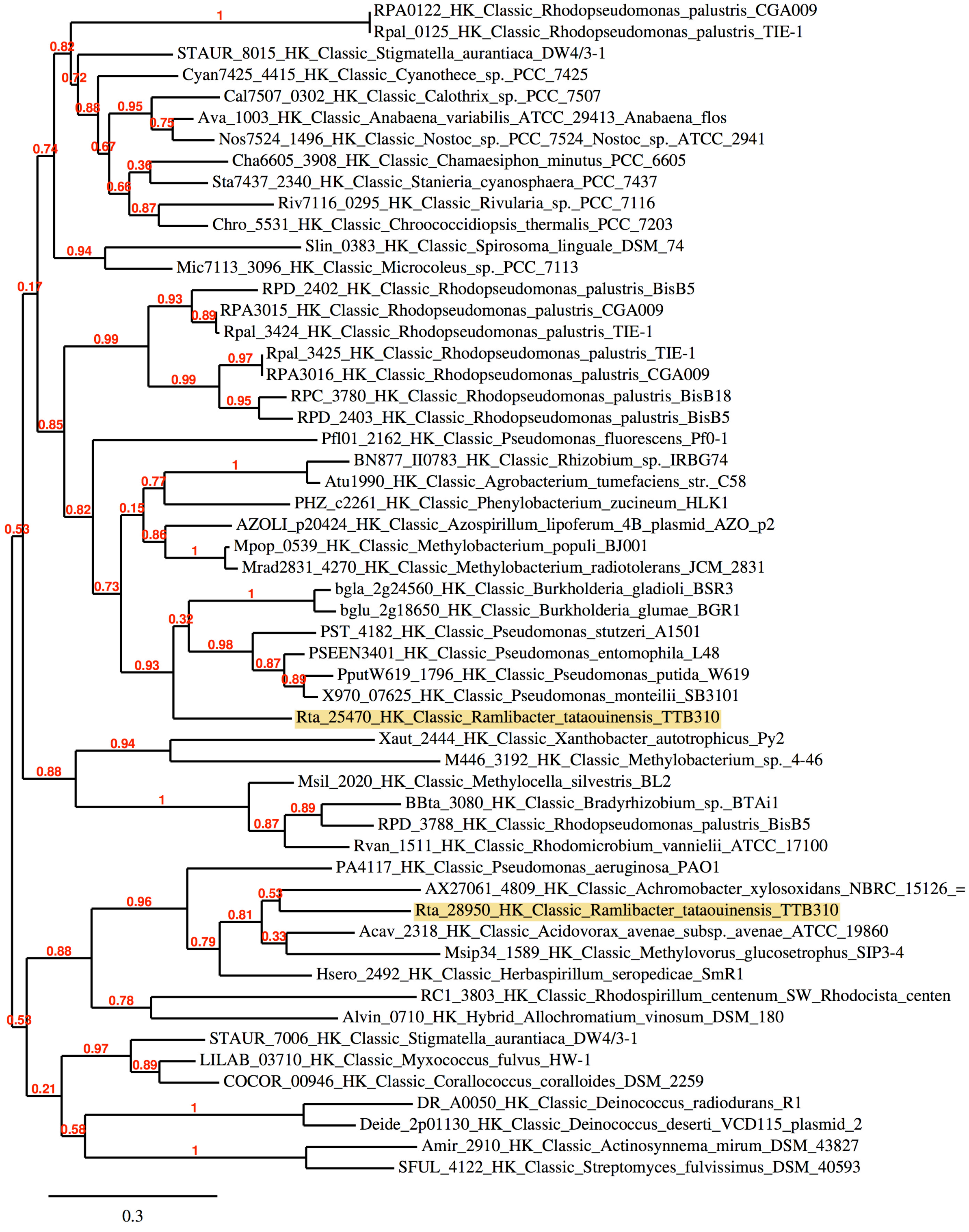
RPA0122_HK_Classic_Rhodopseudomonas_palustris_CGA009
Rpal_0125_HK_Classic_Rhodopseudomonas_palustris_TIE-1
STAUR_8015_HK_Classic_Stigmatella_aurantiaca_DW4/3-1
Cyan7425_4415_HK_Classic_Cyanothece_sp._PCC_7425
Cal7507_0302_HK_Classic_Calothrix_sp._PCC_7507
Ava_1003_HK_Classic_Anabaena_variabilis_ATCC_29413_Anabaena_flos
Nos7524_1496_HK_Classic_Nostoc_sp._PCC_7524_Nostoc_sp._ATCC_2941
Cha6605_3908_HK_Classic_Chamaesiphon_minutus_PCC_6605
Sta7437_2340_HK_Classic_Stanieria_cyanosphaera_PCC_7437
Riv7116_0295_HK_Classic_Rivularia_sp._PCC_7116
Chro_5531_HK_Classic_Chroococcidiopsis_thermalis_PCC_7203
Slin_0383_HK_Classic_Spirosoma_linguale_DSM_74
Mic7113_3096_HK_Classic_Microcoleus_sp._PCC_7113
RPD_2402_HK_Classic_Rhodopseudomonas_palustris_BisB5
RPA3015_HK_Classic_Rhodopseudomonas_palustris_CGA009
Rpal_3424_HK_Classic_Rhodopseudomonas_palustris_TIE-1
Rpal_3425_HK_Classic_Rhodopseudomonas_palustris_TIE-1
RPA3016_HK_Classic_Rhodopseudomonas_palustris_CGA009
RPC_3780_HK_Classic_Rhodopseudomonas_palustris_BisB18
RPD_2403_HK_Classic_Rhodopseudomonas_palustris_BisB5
Pfl01_2162_HK_Classic_Pseudomonas_fluorescens_Pf0-1
BN877_II0783_HK_Classic_Rhizobium_sp._IRBG74
Atu1990_HK_Classic_Agrobacterium_tumefaciens_str._C58
PHZ_c2261_HK_Classic_Phenylobacterium_zucineum_HLK1
AZOLI_p20424_HK_Classic_Azospirillum_lipoferum_4B_plasmid_AZO_p2
Mpop_0539_HK_Classic_Methylobacterium_populi_BJ001
Mrad2831_4270_HK_Classic_Methylobacterium_radiotolerans_JCM_2831
bgla_2g24560_HK_Classic_Burkholderia_gladioli_BSR3
bglu_2g18650_HK_Classic_Burkholderia_glumae_BGR1
PST_4182_HK_Classic_Pseudomonas_stutzeri_A1501
PSEEN3401_HK_Classic_Pseudomonas_entomophila_L48
PputW619_1796_HK_Classic_Pseudomonas_putida_W619
X970_07625_HK_Classic_Pseudomonas_monteilii_SB3101
Rta_25470_HK_Classic_Ramlibacter_tataouinensis_TTB310
Xaut_2444_HK_Classic_Xanthobacter_autotrophicus_Py2
M446_3192_HK_Classic_Methylobacterium_sp._4-46
Msil_2020_HK_Classic_Methylocella_silvestris_BL2
BBta_3080_HK_Classic_Bradyrhizobium_sp._BTAi1
RPD_3788_HK_Classic_Rhodopseudomonas_palustris_BisB5
Rvan_1511_HK_Classic_Rhodomicrobium_vannielii_ATCC_17100
PA4117_HK_Classic_Pseudomonas_aeruginosa_PAO1
AX27061_4809_HK_Classic_Achromobacter_xylosoxidans_NBRC_15126_=
Rta_28950_HK_Classic_Ramlibacter_tataouinensis_TTB310
Acav_2318_HK_Classic_Acidovorax_avenae_subsp._avenae_ATCC_19860
Msip34_1589_HK_Classic_Methylovorus_glucosetrophus_SIP3-4
Hsero_2492_HK_Classic_Herbaspirillum_seropedicae_SmR1
RC1_3803_HK_Classic_Rhodospirillum_centenum_SW_Rhodocista_centen
Alvin_0710_HK_Hybrid_Allochromatium_vinosum_DSM_180
STAUR_7006_HK_Classic_Stigmatella_aurantiaca_DW4/3-1
LILAB_03710_HK_Classic_Myxococcus_fulvus_HW-1
COCOR_00946_HK_Classic_Corallococcus_coralloides_DSM_2259
DR_A0050_HK_Classic_Deinococcus_radiodurans_R1
Deide_2p01130_HK_Classic_Deinococcus_deserti_VCD115_plasmid_2
Amir_2910_HK_Classic_Actinosynnema_mirum_DSM_43827
SFUL_4122_HK_Classic_Streptomyces_fulvissimus_DSM_40593
0.3

FIGURE S2. **Phylogenetic tree of the bacterial bacteriophytochromes identified from all available bacterial genomes**

The two RtBphPs are histidine kinases (sensor part of two-component system) and as indicated in the Materials and Methods section, we opted for an extraction of homologous proteins from the P2CS database (www.p2cs.org). Phylogenetic analysis, using the RtBphPs and their 100-most homologous sequences, was based on the alignment of the photosensory core domain (PCD). When homologous intra-species sequences were detected, only one was kept to make a simplified tree. This tree was generated by using the "*one click mode*" on phylogeny.fr website. The position of the two bacteriophytochromes *Rt*BphP1 (Rta_25470) and *Rt*BphP2 (Rta_28950) are underlined in yellow.

This phylogenetic tree includes bacteriophytochromes identified from complete bacterial genomes, and highlights the presence of previously undescribed putative bacteriophytochromes in several bacterial species (*Pseudomonas fluorescens*, *Pseudomonas stutzeri*, *Pseudomonas putida*, *Burkholderia gladioli*, *Burkholderia glumae*, *Phenylobacterium zucineum*, *Methylobacterium populi*, *Methylobacterium radiotolerans*, *Achromobacter xylosoxidans*, *Methylovorus glucosetrophus*, *Acidovorax avenae*, *Rhodospirillum centenum*, *Allochromatium vinosum*, *Myxococcus fulvus*, *Streptomyces fulvissimus*, and *Xanthobacter autotrophicus*).

FIGURE S3

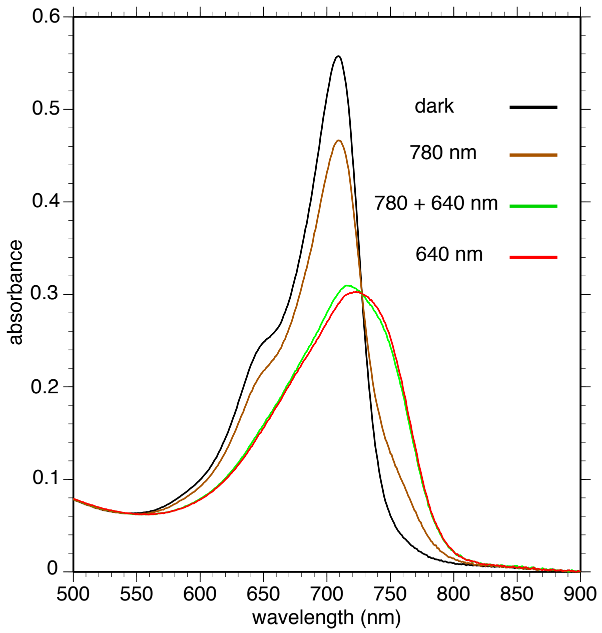

FIGURE S3. **Absorption spectra of recombinant *Rt*BphP1**

Absorption spectra recorded after dark-adaptation (*black line*) and after photoconversion by 780-nm light (*brown line*), 640-nm light (*red line*), or 780-nm + 640-nm light (*green line*).

FIGURE S4

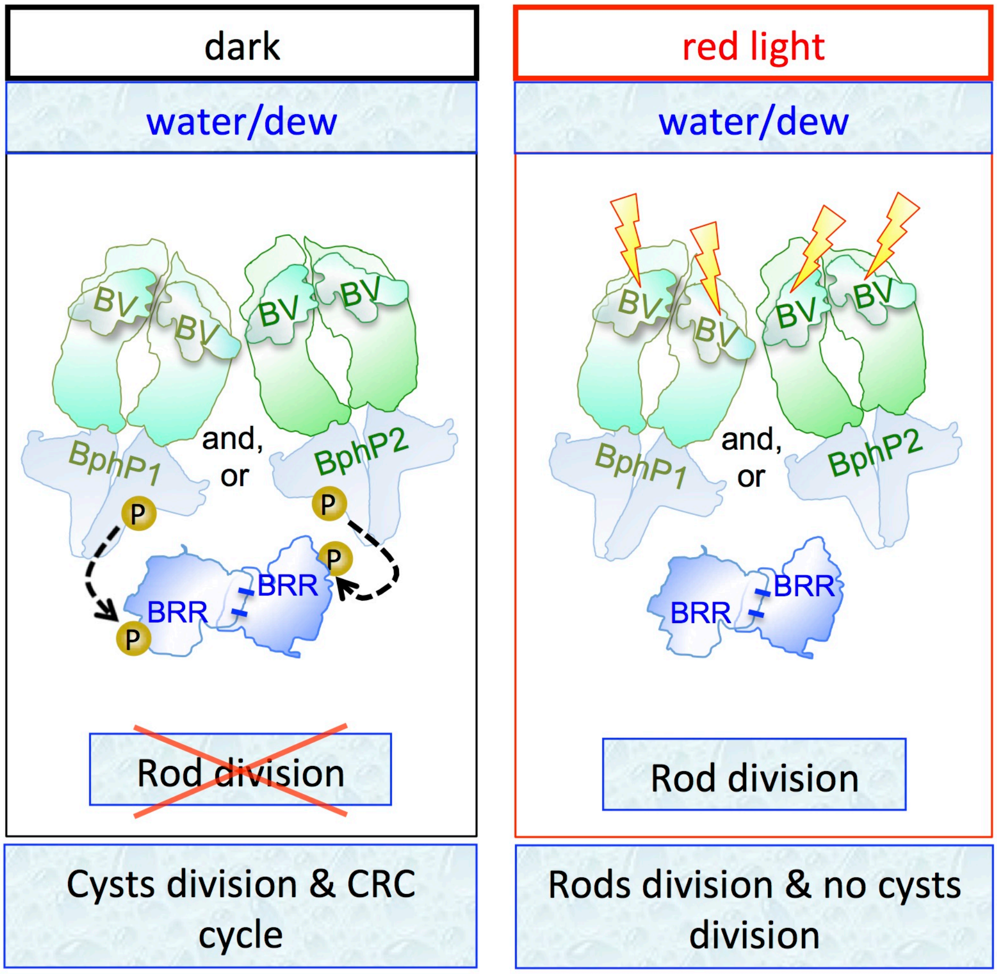

FIGURE S4. **Hypothetical schema of bacteriophytochromes effect on the *Ramlibacter tataouinensis* cell cycle**

In the dark, the cyst-rod-cyst (CRC) cycle and the cyst-cyst (CC) divisions occur whereas the rod-rod (RR) divisions are repressed through the phosphorylation of bacteriophytochromes BphP1 (Rta_25470) and/or BphP2 (Rta_28950), and the cognate bacteriophytochrome response regulator (BRR, Rta_25480). In permissive light, the repression is abolished through the BphPs biliverdine (BV) photoisomerization and allows rod-rod (RR) divisions.
